# Estrogen Abolishes the Repression Role of gga-miR-221-5p Targeting *ELOVL6* and *SQLE* to Promote Lipid Synthesis in Chicken Liver

**DOI:** 10.3390/ijms21051624

**Published:** 2020-02-27

**Authors:** Ding-Ding Zhang, Dan-Dan Wang, Zhang Wang, Yang-Bin Wang, Guo-Xi Li, Gui-Rong Sun, Ya-Dong Tian, Rui-Li Han, Zhuan-Jian Li, Rui-Rui Jiang, Xiao-Jun Liu, Xiang-Tao Kang, Hong Li

**Affiliations:** 1 College of Animal Science and Veterinary Medicine, Henan Agricultural University, Zhengzhou 450002, China; 15736702905@163.com (D.-D.Z.); wdd13938406174@163.com (D.-D.W.); wangzh19930124@163.com (Z.W.); ybwang2008@henau.edu.cn (Y.-B.W.); liguoxi0914@henau.edu.cn (G.-X.L.); grsun2000@126.com (G.-R.S.); ydtian111@163.com (Y.-D.T.); rlhan@henau.edu.cn (R.-L.H.); lizhuanjian@henau.edu.cn (Z.-J.L.); jrrcaas@163.com (R.-R.J.); xiaojun.liu@henau.edu.cn (X.-J.L.); xtkang2001@263.net (X.-T.K.); 2 Henan Innovative Engineering Research Center of Poultry, Zhengzhou 450002, China; 3 International Joint Research Laboratory for Poultry Breeding of Henan, Zhengzhou 450002, China

**Keywords:** gga-miR-221-5p, *ELOVL6*, *SQLE*, lipid metabolism, chicken, estrogen

## Abstract

Few studies have been conducted regarding the biological function and regulation role of gga-miR-221-5p in the liver. We compared the conservation of miR-221-5p among species and investigated the expression pattern of gga-miR-221-5p, validating the direct target genes of gga-miR-221-5p by dual luciferase reporter assay, the biological function of gga-miR-221-5p in the liver was studied by gga-miR-221-5p overexpression and inhibition. Furthermore, we explored the regulation of gga-miR-221-5p and its target genes by treatment with estrogen and estrogen antagonists in vivo and in vitro. The results showed that miR-221-5p was highly conserved among species, expressed in all tested tissues and significantly downregulated in peak-laying hen liver compared to pre-laying hen liver. Gga-miR-221-5p could directly target the expression of elongase of very long chain fatty acids 6 (*ELOVL6)* and squalene epoxidase (*SQLE*) genes to affect triglyceride and total cholesterol content in the liver. 17β-estradiol could significantly inhibit the expression of gga-miR-221-5p but promote the expression of *ELOVL6* and *SQLE* genes. In conclusion, the highly conservative gga-miR-221-5p could directly target *ELOVL6* and *SQLE* mRNAs to affect the level of intracellular triglyceride and total cholesterol. Meanwhile, 17β-estradiol could repress the expression of gga-miR-221-5p but increase the expression of *ELOVL6* and *SQLE*, therefore promoting the synthesis of intracellular triglyceride and cholesterol levels in the liver of egg-laying chicken.

## 1. Introduction

MicroRNAs (miRNAs) are small, endogenous, non-coding single-strand RNAs that post-transcriptionally regulate gene expression by binding to the 3′ untranslated regions (3′UTR) of potentially hundreds of mRNAs. miRNAs are about 22 nucleotides (nt) in length [1,2], and the 2nd–8th nt of the 5′ end of miRNA, named the seed region, are crucial for recognizing and binding to target genes [3,4]. miRNAs regulate their target mRNAs by mRNA molecule degradation or translation inhibition [5,6].

Increasingly many investigations suggest that miRNAs are involved in various biological processes. Recent studies report that miRNAs exert a biological function in metabolic and endocrine pathways [7] and adipogenesis [8,9]. Emerging evidence shows that miRNAs act as key regulators for hepatic lipid metabolism. For example, the liver-specific miR-122 in mammals is abundantly expressed in liver [10] and can control the hepatic fatty acids and cholesterol synthesis by repressing the expression of mRNAs involved in the cholesterol biosynthesis pathway [11]. miR-33 can regulate the biological metabolism process of the liver by affecting cholesterol efflux and high-density lipoprotein metabolism [12]. Gga-miR-22-3p and gga-miR-101-2-5p are involved in the hepatic lipid metabolism by targeting the key factors *Elovl6* and Apolipoprotein B (*ApoB*) of the lipid metabolism pathway [13,14]. The differential expression of miRNAs has been reported in the liver tissues of pre-laying and peak-laying hens [15], and obese and non-obese human individuals [16,17]. Most of the studies about miRNA functions are mainly focused on mammals, however, the action mechanisms of most miRNAs in chicken are unclear.

The liver plays a key role in lipid metabolism. Especially in poultry, the liver is the hub of fatty acid synthesis and lipid circulation through lipoprotein synthesis [18]. With the onset of egg production, estrogen shifts hepatocytic lipoprotein production to assemble into triacylglycerol-rich yolk very low density lipoprotein (VLDL) [19]. Egg laying is the most important economic trait in hens, and this physiological process is highly influenced by chicken liver function. Our recent study displayed that, compared with pre-laying (20 weeks) hens, gga-miR-221-5p was significantly downregulated in the liver tissue of peak-laying (30 weeks) hens [15] and gga-miR-221-5p was significantly downregulated in the liver tissue of estrogen-treated hens (10 weeks) compared with 10-week-old hens’ livers (unpublished data) [20]. Therefore, it is of great significance to investigate the biological function of gga-miR-221-5p, which might be relevant to hepatic lipid metabolism.

To date, most of the studies about miR-221-5p have focused on diseases in mammals and poultry. MiR-221-5p is located in the intergenic region and is mainly reported to be a known player in oncogenesis, affecting the proliferation and behavior of various cell types [21,22,23,24]. It is suggested that miR-221 also participates in glucose/fat metabolism. miR-221 may contribute to the development of the insulin resistance that typically accompanies obesity by regulating the peroxisome proliferator activated receptor (*PPAR*) signaling pathway and directly down-regulating adiponectin receptor 1 (*ADIPOR1*) and ETS proto-oncogene 1, transcription factor (*ETS1*) [25]. Obese mice show high miR-221 levels and low *AdipoR1* levels [26]. MiR-221-5p is suggested to act as a Marek’s disease tumor suppressor [27]. The expression of miR-221 is significantly upregulated in ALV-infected chicken [28]. In addition, gga-miR-221 is implied to be a tumor formation-relevant gene in chicken [29]. However, little is known about the regulation role of gga-miR-221 in the chicken lipid metabolism. Therefore, in this study, we have systematically investigated the biological role and the regulation role of gga-miR-221-5p in the lipid metabolism of chicken.

## 2. Results

### 2.1. Conservative Analysis of miR-221-5p Among Species

**Figure 1:**
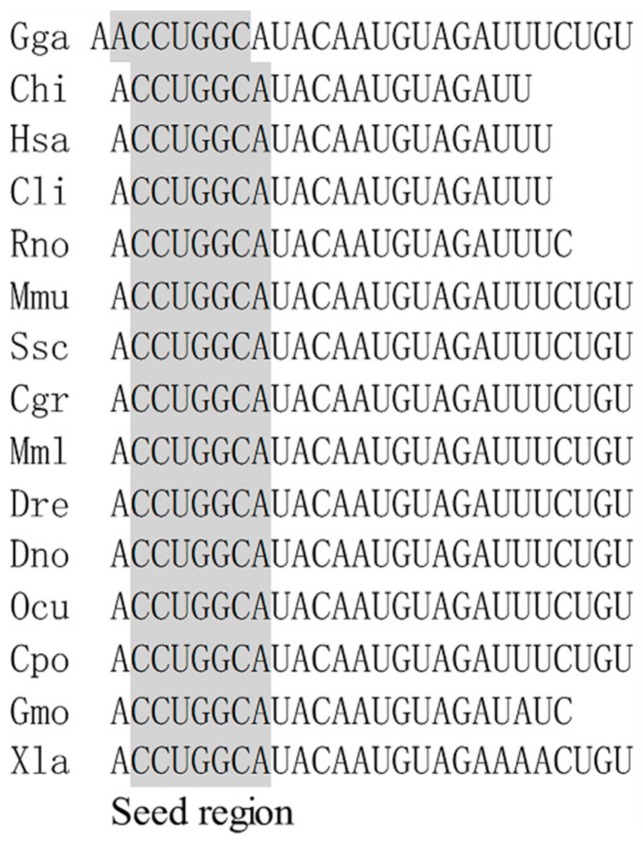
Alignment of miR-221-5p mature sequence among species. Note: Gga: *Gallus gallus*; Chi: *Capra hircus*; Has: *Homo sapiens*; Cli: *Columba livia*; Rno: *Rattus norvegicus*; Mmu: *Mus musculus*; Ssc: *Sus scrofa*; Cgr: *Cricetulus griseus*; Mml: *Macaca mulatta*; Dre: *Danio rerio*; Dno: *Dromaius novaehollandiae*; Ocu: *Oryctolagus cuniculus*; Cpo: *Crocodylus porosus*; Gmo: *Gadus morhua*; Xla: *Xenopus laevis*.

The mature miR-221-5p sequences derived from different species were collected, and the conservatism of this among species was aligned. The alignment result showed that both the mature miR-221-5p sequences and seed regions were highly conserved among *Gallus gallus*, *Capra hircus*, *Homo sapiens*, *Columba livia*, *Rattus norvegicus*, *Mus musculus*, *Sus scrofa*, *Cricetulus griseus*, *Macaca mulatta*, *Danio rerio*, *Dromaius novaehollandiae*, *Oryctolagus cuniculus*, and *Crocodylus porosus*. Only one or three bases in the 3’ end of mature sequences were not matched between the two species *Gadus morhua* and *Xenopus laevis* and the others (Figure 1).

### 2.2. Spatiotemporal Expression Characteristics of gga-miR-221-5p

**Figure 2:**
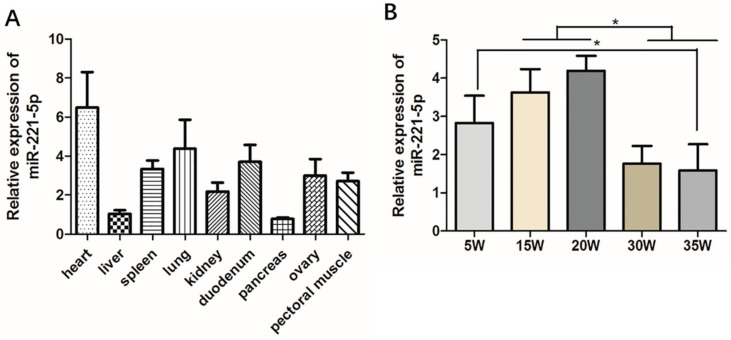
Spatiotemporal expression profile of gga-miR-221-5p. (**A**) The relative expression of gga-miR-221-5p in different tissues. (**B**) The relative expression of gga-miR-221-5p in different liver stages. *β-actin* was used as an internal reference gene to estimate the relative expression of mRNA. *U6* was used as an internal reference gene to estimate the relative expression of miRNA. Data are represented as mean ± SD (*n* = 6). * means 0.01 < *p* < 0.05.

In order to initially explore the basic biological expression characteristics of gga-miR-221-5p, tissue expression distributions of gga-miR-221-5p were detected in various tissues of 30-week-old chickens. It was shown that gga-miR-221-5p was broadly expressed in different tissues (Figure 2A). The relative expression level of gga-miR-221-5p was highest in the heart and lowest in the liver and pancreas. Spatio-temporal expression pattern of gga-miR-221-5p in livers at different stages was detected (Figure 2B). The results showed that the relative expression levels of gga-miR-221-5p in the livers of chicken at the age of 15 and 20 weeks were significantly higher than at 30 and 35 weeks (*p* < 0.05). The expression difference was not significant among 5, 15 and 20 weeks (*p* > 0.05). There was also no significant difference existence between 30 and 35 weeks (*p* > 0.05).

### 2.3. Screening of gga-miR-221-5p Candidate Target Genes

**Figure 3:**
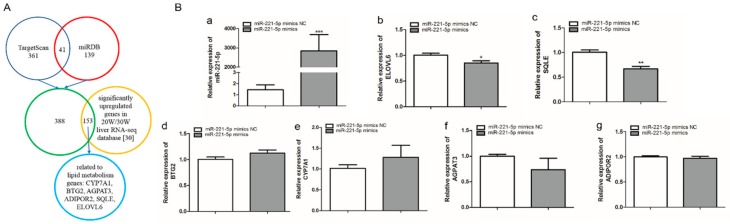
Screening of gga-miR-221-5p candidate target genes. (**A)** The selection process of the gga-miR-221-5p target genes. (**B)** Relative expression levels of gga-miR-221-5p and candidate target genes after gga-miR-221-5p mimics treatment for 48 h. * means 0.01 < *p* < 0.05, ** means 0.001 < *p* < 0.01, *** means *p* < 0.0001.

402 and 180 target genes of gga-miR-221-5p were predicted by TargetScan and miRDB online software, respectively, taking the union set of the two online software predicted target genes to obtain 541 potential target genes; these genes were intersected with the significantly upregulated genes in the 20-week and 30-week liver RNA-seq database [30], and 153 potential target genes were obtained, of which BTG anti-proliferation factor 2 (*BTG2*), cytochrome P450 family 7 subfamily A member 1 (*CYP7A1*), 1-acylglycerol-3-phosphate O-acyltransferase 3 (*AGPAT3*), *ADIPOR2*, *SQLE,* and *ELOVL6* genes related to lipid metabolism were selected as potential target genes for further validation (Figure 3A). To further screen the possible targets, gga-miR-221-5p mimics was used to transfect LMH chicken hepatoma cells. According to the qRT-PCR analysis result, compared with the miR-221-5p mimics negative control (miR-221-5p mimic NC) treatment group, the relative expression of gga-miR-221-5p was significantly increased in the miR-221-5p mimics treated group (*p* < 0.0001). Only the mRNA relative expression levels of *SQLE* and *ELOVL6* were significantly downregulated in the miR-221-5p mimics treated group (*p* < 0.05 or *p <* 0.01), and there was no significant alteration in *BTG2*, *CYP7A1*, *AGPAT3*, and *ADIPOR2* compared to the miR-221-5p mimic NC group (*p* > 0.05; Figure 3B). 

**Figure 4:**
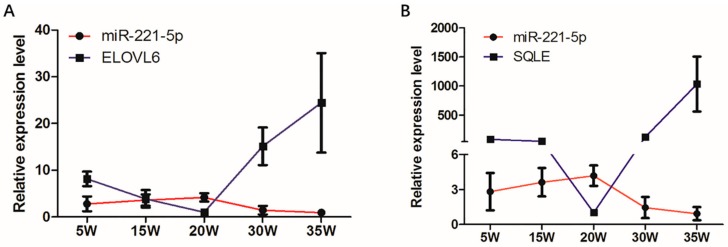
Expression of gga-miR-221-5p and candidate target genes in different liver stages. (**A**) Expression of gga-miR-221-5p and *ELOVL6* in different liver stages. (**B**) Expression of gga-miR-221-5p and *SQLE* in different liver stages. *β-actin* was used as an internal reference gene to estimate the relative expression of mRNA. *U6* was used as an internal reference gene to estimate the relative expression of miRNA. Data are represented as mean ± SD (*n* = 6).

**Figure 5:**
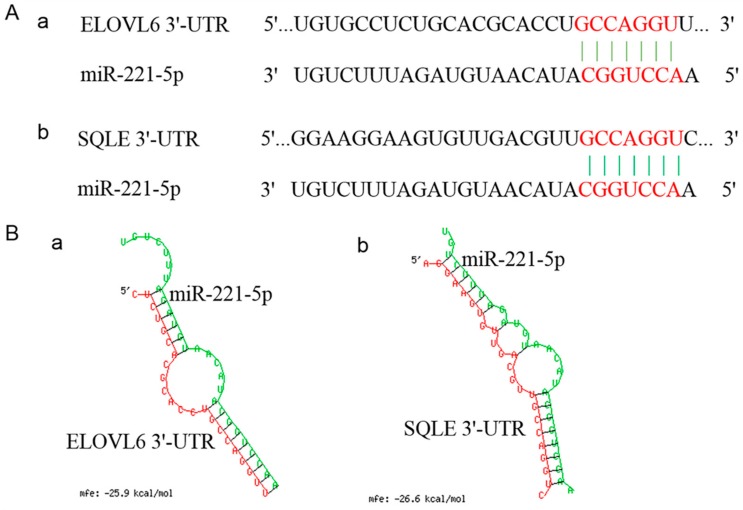
Sequencing information of *ELOVL6*’s and *SQLE*’s 3′ untranslated regions (3′UTR) binding to the gga-miR-221-5p seed region and the prediction of its free energy. (**A**) Sequencing information of *ELOVL6*’s and *SQLE*’s 3′UTR binding to the gga-miR-221-5p seed region. (**B**) Free energy information of *ELOVL6*’s and *SQLE*’s 3′UTR binding to gga-miR-221-5p.

The spatio-temporal expression analysis displayed that the expression modes of *ELOVL6* and *SQLE* in the chicken livers at different stages were completely opposite to that of gga-miR-221-5p (Figure 4). In addition, the sequences located in the 3′UTR of *ELOVL6* and *SQLE* genes where the gga-miR-221-5p seed region was recognized are shown in Figure 5A. The MFE of gga-miR-221-5p binding to the 3′UTR of *ELOVL6* and *SQLE* was −25.9 kcal/mol and −26.6 kcal/mol, respectively (Figure 5B), indicating that the complexes of gga-miR-221-5p binding with *ELOVL6* and *SQLE* occurred with high stability. It was suggested that *SQLE* and *ELOVL6* might be the possible direct target genes of gga-miR-221-5p.

### 2.4. Verification of the Interaction Relationship between gga-miR-221-5p and Potential Target Genes

**Figure 6:**
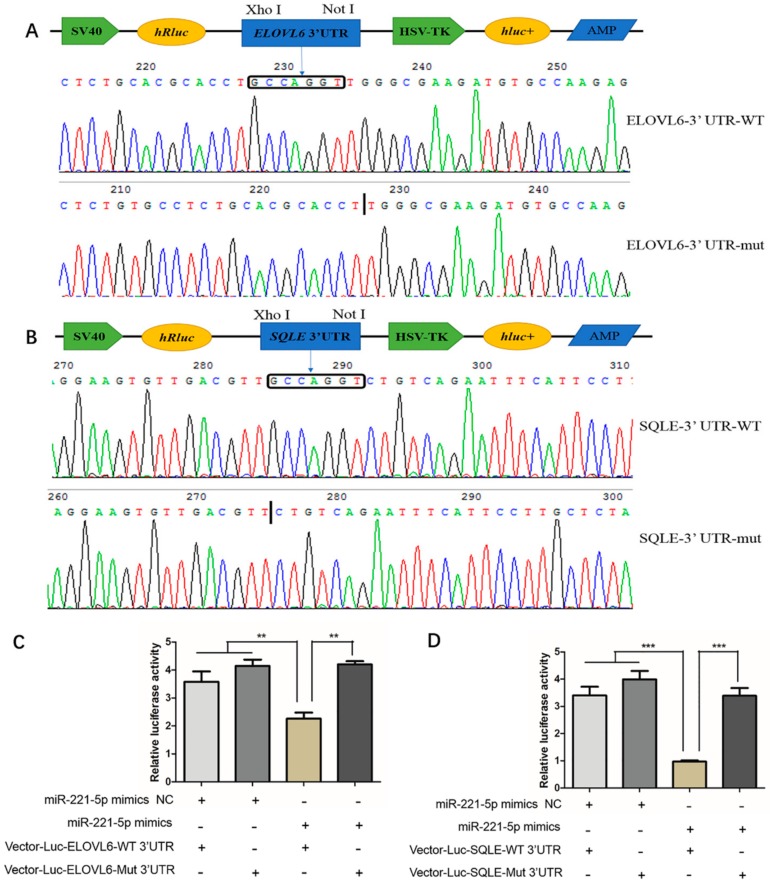
Validation of gga-miR-221-5p and the *ELOVL6* and *SQLE* targeting relationship. (**A**,**B**) Recombinant plasmid sequencing. (**C**,**D**) Dual luciferase reporter assay analysis of the target relationship of gga-miR-221-5p with *ELOVL6* and *SQLE* in LMH chicken hepatoma cells. The long box □ indicates the position of the gga-miR-221-5p seed region in combination with the *ELOVL6* and the *SQLE* gene 3′UTR, the black vertical line indicates the position of the *ELOVL6* and *SQLE* gene 3′UTR deletion binding to the gga-miR-221-5p seed region. WT indicates wild, Mut indicates mutation, + indicates added, - indicates not added. Data are represented as mean ± SD (*n* = 6). ** means 0.001 < *p* < 0.01, *** means *p* < 0.0001.

To verify whether gga-miR-221-5p interacts with *ELOVL6* and *SQLE* genes, we constructed Vector-Luc-ELOVL6-WT 3′UTR, Vector-Luc-ELOVL6-Mut 3′UTR, Vector-Luc-SQLE-WT 3′UTR, and Vector-Luc-SQLE-Mut 3′UTR for the verification of the interaction between gga-miR-221-5p and the two potential target genes (Figure 6A,B), and the dual luciferase reporter assay was conducted in LMH chicken hepatoma cells. The results showed that, when co-transfected with gga-miR-221-5p mimics and Vector-Luc-ELOVL6-WT 3′UTR, the relative fluorescence activity was significantly inhibited (*p* < 0.01), and the inhibition was abolished in the gga-miR-221-5p mimics and Vector-Luc-ELOVL6-Mut 3′UTR co-treated group. No significant difference was observed among gga-miR-221-5p mimics and Vector-Luc-ELOVL6-Mut 3′UTR co-treated, gga-miR-221-5p mimics NC and Vector-Luc-ELOVL6-WT 3’UTR co-treated, gga-miR-221-5p mimics NC and Vector-Luc-ELOVL6-Mut 3’UTR co-treated groups (*p* > 0.05). Furthermore, the same interaction relationship was also found between gga-miR-221-5p and the *SQLE* gene (Figure 6C,D).

### 2.5. Effect of gga-miR-221-5p on Intracellular Triglyceride and Total Cholesterol Content

**Figure 7:**
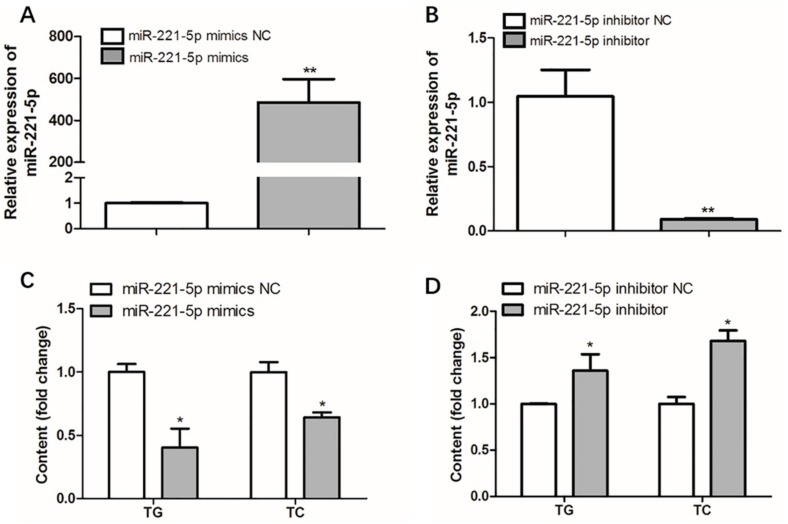
Effect of gga-miR-221-5p overexpression and inhibition on intracellular triglycerides (TG) and cholesterol (TC). (**A**,**B**) The expression of gga-miR-221-5p under the gga-miR-221-5p overexpression and inhibition in LMH chicken hepatoma cells, respectively. (**C**,**D**) Changes of TG and TC contents under gga-miR-221-5p overexpression and inhibition in LMH chicken hepatoma cells, respectively. Data are represented as mean ± SD (*n* = 6). * means 0.01 < *p* < 0.05, ** means 0.001 < *p* < 0.01.

To measure the effect of gga-miR-221-5p on intracellular triglyceride (TG) and total cholesterol (TC) levels, over-expression and interference assays of gga-miR-221-5p were carried out, respectively. The over-expression experiment result showed that the expression level of gga-miR-221-5p was upregulated by approximately 500-fold compared with the control group (miR-221-5p mimics NC group). Interestingly, the levels of intracellular TG and TC were significantly downregulated in the miR-221-5p mimics group compared with the miR-221-5p mimics NC group (*p* < 0.05). The interference experiment showed that the expression level of gga-miR-221-5p was downregulated by approximately 90%, and the contents of intracellular TG and TC were significantly upregulated in the miR-221-5p inhibitor group than the miR-221-5p inhibitor NC group (*p* < 0.05; Figure 7).

### 2.6. 17β-Estradiol Repressed the Expression of gga-miR-221-5p and Promoted the Expression of Target Genes in Chicken Primary Hepatocytes 

**Figure 8:**
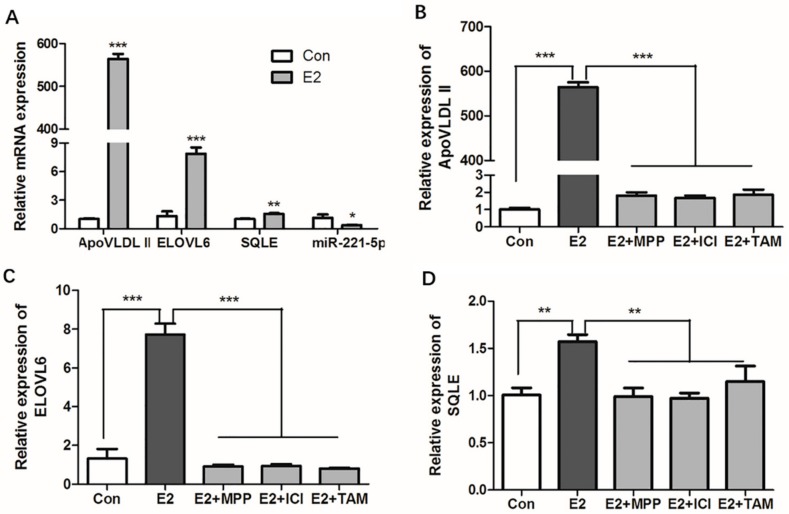
Effects of estrogen and estrogen antagonists on the expression of *ApoVLDL II*, gga-miR-21-5p, *ELOVL6,* and *SQLE* gene in vitro. (**A**) Effects of estrogen on the expression of *ApoVLDL II*, gga-miR-21-5p *ELOVL6,* and *SQLE* gene in vitro. (**B**–**D**) Effects of estrogen and estrogen antagonists co-treated on the expression of *ApoVLDL II*, *ELOVL6,* and *SQLE* gene in vitro. Con indicates control group, E2 indicates 17β-estradiol (100nM) treated group, E2 + MPP indicates 17β-estradiol (100 nM) and MPP co- treated group, E2 + ICI indicates 17β-estradiol (100 nM), and ICI 182,780 co-treated group, E2 +TAM indicates 17β-estradiol (100 nM), and tamoxifen co-treated group, respectively. *β-actin* was used as an internal reference gene. Data are represented as mean ± SD (*n* = 6). * means 0.01 < *p* < 0.05, ** means 0.001 < *p* < 0.01, *** means *p* < 0.0001.

Estrogen is an important regulator in lipid metabolism, and 17β-estradiol was used to stimulate the primary hepatocytes. The results showed that the estrogen response marker gene, apovitellenin very low density lipoprotein Ⅱ (*ApoVLDL II*) [31], was significantly upregulated by 17β-estradiol (*p* < 0.0001) in chicken primary hepatocytes, which indicated the estrogen exerted effects in primary hepatocytes, and an in vitro estrogen induction model was successfully constructed. Compared with the control group, gga-miR-221-5p was significantly repressed (*p* < 0.05); in contrast, the expression of *ELOVL6* and *SQLE* was significantly promoted by 17β-estradiol (*p* < 0.0001 or *p* < 0.001) in chicken primary hepatocytes (Figure 8A).

The classic model of estrogen action requires that the estrogen receptor (ER) activates gene expression. To know which ER subtype estrogen acts through, chicken primary hepatocytes were treated with different ER antagonists. The literature states that *ApoVLDL II* gene is regulated by ERα [31], and the expression of the *ApoVLDL II* gene under the estrogen antagonist treatment results indicated that estrogen antagonist treatment experiment was successful (Figure 8B). The results showed that 17β-estradiol could significantly increase the mRNA expression levels of *ELOVL6* and *SQLE* in primary hepatocytes (*p* < 0.0001 or *p* < 0.001). Compared with the 17β-estradiol treatment group, the expressions of *ELOVL6* and *SQLE* were significantly decreased in the ER antagonist treatment groups (*p* < 0.0001 or *p* < 0.001). The expression levels of the genes returned to the control level in the ER subtype antagonist-treated groups (*p* > 0.05; Figure 8C,D). Online software predicted results showed that atypical estrogen responsive elements (ERE) binding sites (GGGCACTAAGACC and GCTCACATTGTCC) were found in the -5000 bp promoter region of *ELOVL6* and *SQLE* genes, respectively, and the *SQLE* gene was also found in our chip database established by estrogen receptor alpha chromatin immunoprecipitation experiment (unpublished data) [20]. Since methyl-piperidino-pyrazole (MPP) is the antagonist of estrogen receptor alpha (ERα), the ICI 182,780 and tamoxifen (TAM) are the antagonists of ERα and estrogen receptor beta (ERβ). The expression of *ELOVL6* and *SQLE* in 17β-estradiol and ICI 182,780 co-treated, 17β-estradiol and tamoxifen co-treated groups did not decrease further compared with the 17β-estradiol and MPP co-treated group. This revealed that estrogen could activate the expression of *SQLE* via ERα, while estrogen might activate the expression of *ELOVL6* via other estrogen receptors or intermediates.

### 2.7. Effect of 17β-Estradiol on gga-miR-221-5p and the Target Genes in Chicken Liver

**Figure 9:**
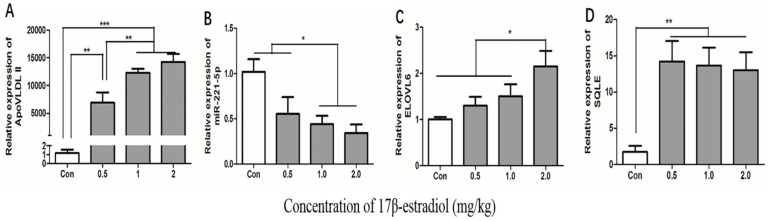
Effects of estrogen on the expression of *ApoVLDL II*, gga-miR-221-5p, *ELOVL6,* and *SQLE* gene in vivo. Con indicates control group. *β-actin* was used as an internal reference gene. *U6* was used as an internal reference gene to estimate the relative expression of miRNA. Data are represented as mean ± SD (*n* = 8). * means 0.01 < *p* < 0.05, ** means 0.001 < *p* <0.01, *** means *p* < 0.0001.

To estimate whether the 17β-estradiol acted similarly on the expression of gga-miR-221-5p and the target genes in vivo as in vitro, chickens were treated with different doses of 17β-estradiol through intramuscular injection. The effect of 17β-estradiol on the expressions of gga-miR-221-5p and the target genes in the liver of chickens were detected by using qRT-PCR (Figure 9). The *ApoVLDL II* expression level, a marker gene [31], was significantly increased in a dose-dependent manner under the action of estrogen, indicating that we successfully constructed an estrogen-treated biological model in vivo. The expression of gga-miR-221-5p was significantly decreased in the 17β-estradiol 1.0 mg/kg and 2.0 mg/kg treated groups (*p* < 0.05). Conversely, the relative expression levels of *ELOVL6* were significantly upregulated in the 2.0 kg/mg 17β-estradiol group (*p* < 0.05), and the relative expression levels of *SQLE* were significantly upregulated in the 17β-estradiol group (*p* < 0.01). 

## 3. Discussion

Numerous studies have shown that miRNAs, as a conserved endogenous regulator in vivo, participate in various metabolic processes of organisms [32,33]. In this study, it was found that the mature sequence of miR-221-5p was relatively highly conserved among species, suggesting that miR-221-5p played an important biological function among species. It has been reported that hsa-miR-221-5p regulates the proliferation and metastasis of prostate cancer cells by targeting the expression of the suppressor of cytokine signaling 1 (*SOCS1*) gene [34]. The analysis of miRNA transcriptomes in Marek’s disease (MD) lymphoma showed that gga-miR-221-5p was significantly downregulated in MD lymphoma, and gga-miR-221-5p inhibited the proliferation of MDV-transformed lymphoid cell line (MSB1), suggesting that gga-miR-221-5p had an anti-cancer effect [27]. The expression profiles of gga-miR-221-5p in different tissues of chicken at the age of 30 weeks showed that it was expressed in a broad-spectrum in various tissues, indicating that gga-miR-221-5p might exert certain biological effects in various tissues of chicken as in other species. The expressions of the liver at different stages showed that the expression level of gga-miR-221-5p at the peak-laying stage was significantly lower than that of the pre-laying stages. The most physiological difference between the Lushi chickens at the age of 20 weeks and 30 weeks was in whether they laid or not; therefore, this further suggests that gga-miR-221-5p might be involved in the chicken egg-laying related processes.

As is well known, miRNAs are involved in different biological processes by regulating the expression of target genes at the post-transcriptional level by binding to the target gene 3′UTR [5,6]. One miRNA may regulate the expression of multiple target genes, and one gene may also be regulated by multiple miRNAs [35]. Gga-miR-221-5p was demonstrated to directly interact with *ELOVL6* and *SQLE* genes by binding to the 3′UTR of mRNAs with high stability, and controlling the expression of *ELOVL6* and *SQLE* at the post-transcriptional level. The *ELOVL6* gene, a member of the fatty acyl elongase (ELOVL) gene family, is the only enzyme that participated in de novo lipogenesis and is responsible for the final step of endogenous saturated fatty acid synthesis, which plays an important role in the fatty acid synthesis pathway [36]. It was found that the *ELOVL6* promoter region has a QTL polymorphism site that can regulate the composition of porcine fatty acid [37]. Studies in poultry have found that single nucleotide polymorphisms in the *ELOVL6* gene are associated with the deposition of subcutaneous fat [38]. SQLE located on the endoplasmic reticulum is the first oxygenation step in the biosynthesis of sterols and is considered to be one of the key enzymes in this pathway, which has an important impact on the flux synthesis of cholesterol [39]. An abnormal expression of the *SQLE* gene in abnormal cholesterol metabolism cells has been reported, such as prostate cancer, breast cancer, and lung cancer [40,41,42]. The function loss or gain assays of gga-miR-221-5p showed a promotion or inhibition role on the content of intracellular TG and TC, respectively. Our result demonstrated that gga-miR-221-5p participated in the lipid metabolism pathway by regulating the expression of the *ELOVL6* and *SQLE* genes.

The relative expression between gga-miR-221-5p and *ELOVL6* or *SQLE* in the liver at different stages showed a negative correlation, which revealed that gga-miR-221-5p might be involved in different physiological periods by targeting the *ELOVL6* and *SQLE* genes in a chicken. It was interested to found that gga-miR-221-5p was significantly downregulated at the egg-laying stages compared with pre-laying stages. Studies report that, with the arrival of sexual maturity, estrogen levels gradually rise and reach the highest level at sexual maturity (i.e., when the first egg is laid) [43,44]. During the egg-laying stage, under the promotion of estrogen, a large number of neutral lipids including triacylglycerols, cholesteryl esters, and free fatty acids are synthesized in the liver, then assembled into very-low-density lipoprotein (VLDL) and vitellogenin particles and transported to ovary to generate the egg-yolk precursors through blood circulation [45,46]. Genes involved in this lipid synthesis process, including *ELOVL6* related to fatty acids and *SQLE* related to cholesterol, were upregulated by estrogen at the genome level, while estrogen repressed the expression of gga-miR-221-5p, which was proved by in vitro and in vivo trial. Moreover, we found that the TG and TC contents were also dramatically increased under the estrogen treatment in chicken [47], and our results were consistent with previous studies [48]. Meanwhile, it is well known that liver X receptor alpha (LXR-α) is also an important regulators of liver lipid metabolism. LXRα is functional when heterodimerized with retinoid x receptors (RXRs), and regulated the transcription of their target genes by binding to specific response elements (LXREs) that contain a hexametric nucleotide direct repeat spaced by four bases (DR4) [49]. A previous study showed that LXR gene knockout mice blocked cholesterol metabolism by downregulating ATP-binding cassette A1 (*ABCA1*) gene expression [50]. In many species, the activation of LXR-α can promote the biosynthesis of cholesterol and fatty acids, key genes for lipid metabolism such as *SREBP-1c*, Acetyl-CoA carboxylase (*ACC*) and fatty acid synthase (*FASN*) gene expression levels are significantly upregulated [51,52,53]. While, LXR-α was not significantly changed in our databases from the liver transcriptome data of the pre- and peak-laying hens [30], and the liver transcriptome data of estrogen-treated hens and untreated hens (unpublished data) [20]. It was suggested that LXR-α might be not involve in the estrogen signal pathway.

**Figure 10:**
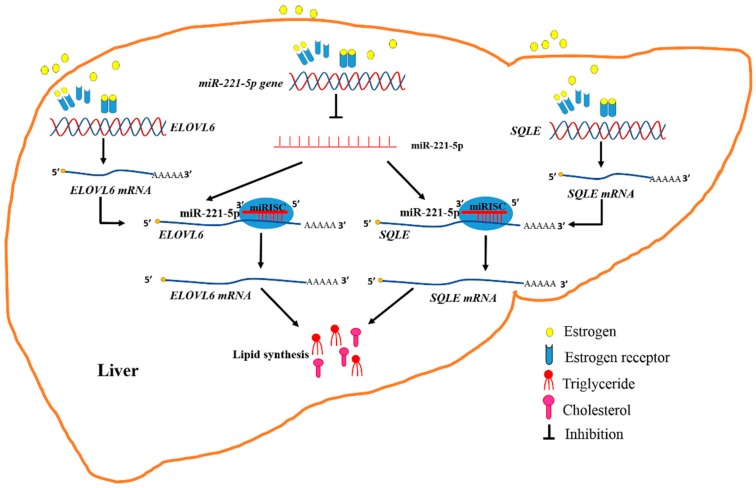
Diagram illustrating the underlying mechanism by which that estrogen and miR-221-5p target *ELOVL6* and *SQLE* to participate in chicken liver lipid metabolism.

Therefore, it was determined that estrogen abolished the inhibitory effect of gga-miR-221-5p on targeting *ELOVL6* and *SQLE* at post-transcriptional levels during the egg-laying period; meanwhile, estrogen promoted the expression of *ELOVL6* and *SQLE*, which resulted in a dramatic increase in TG and TC content in chicken (Figure 10). Generally, estrogen regulates gene expression by signaling through intracellular hormone-specific estrogen receptors (ERα and ERβ). The canonical model for ER-mediated regulation of gene expression involves the direct binding of dimeric ER to DNA sequences known as estrogen response elements (EREs) or the AP1 enhancer element [54,55,56]. If estrogen signaling at a classical ERE, both ERα and ERβ showed the same transactivation profiles. However, if estrogen signaling at the AP1 element, ERα and ERβ would respond differently. The ligand-induced responses with ERβ at an AP1 site showed a negative transcriptional regulation by the natural hormone. Our research found that estrogen promoted the expression of the *SQLE* gene through ERα, but estrogen might promote the expression of the *ELOVL6* gene through the mediation of intermediates. The expression of gga-miR-221-5p was downregulated by estrogen, and an AP1-binding site existed in the −5000 bp region upstream of pri-miR-221-5p (TGAGTCA). It implied that estrogen-binding ERβ might form a complex with Jun/Fos, and binding to the AP1 site, thereby ultimately inhibiting the formation of gga-miR-221-5p. In addition, some other regulation mechanism would be also possible. For example, it was also reported that some other regulators such as long non-coding RNA (lncRNA) controlled by estrogen, and lncRNA could sponge miRNAs and interfere with miRNA expression [57]. Therefore, the regulation mechanisms of estrogens interfere with miR-221-5p expression are complex, and needed to be studied further in the future.

To date, this is the first evidence of the biological function of gga-miR-221-5p controlling the lipid metabolism in the liver of egg-laying chicken. In addition, human lipid metabolism diseases are affected by many factors including lncRNA [58], miRNA [59], transcription factors [60], leucine deficiency [61], etc., and this has always been a hot topic in human research. The literature showed that gga-miR-33 can participate in lipid metabolism by targeting fat mass and obesity-associated (*FTO*) genes associated with obesity in chicken liver [1], and miR-33 can regulate cholesterol and fatty acid metabolism in mammals (humans and mice), which corresponds to the function of the host gene sterol regulatory element binding transcription factor 2 (*SREBP2*), which can regulate the synthesis and uptake of triglycerides and cholesterol [62,63], it can be seen that miR-33 functions similarly in chickens and mammals (humans and mice). Other studies have shown that, in human and mouse liver cells, the overexpression of miR-34a can inhibit the expression of hepatocyte nuclear factor 4 alpha (*HNF4α*) and increase the accumulation of TG. The knockdown of miR-34a can inhibit lipid accumulation and reduce liver cell steatosis [64]. In chicken liver, miR-34a-5p promotes the accumulation of TG by regulating the expression of long-chain acyl-CoA synthetase 1 (*ACSL1*), and it has a function similar to that of human and mouse hepatocytes, thus achieving the positive regulation of fatty acid synthesis [65]. Interestingly, miR-221-5p was highly conserved among species including mammals, and gga-miR-221-5p was involved in liver lipid metabolism; our study results may also provide a valuable reference for humans in lipid study.

In summary, the highly conserved gga-miR-221-5p was proved to directly target *ELOVL6* and *SQLE* mRNAs to affect the level of intracellular triglyceride and total cholesterol in LMH chicken hepatoma cells. Meanwhile, 17β-estradiol could repress the expression of gga-miR-221-5p but increase the expression of *ELOVL6* and *SQLE* mRNAs, thereby promoting the synthesis of intracellular triglyceride and cholesterol levels in the liver of egg-laying chickens. 

## 4. Materials and Methods

### 4.1. Animal Ethics

Animal experiments were performed in accordance with protocols approved by the Institutional Animal Care and Use Committee (IACUC) of Henan Agricultural University, China (Permit Number: 11-0085; Date: 13 June 2011).

### 4.2. Treatment and Sample Collection 

Chinese, locally breed Lushi green-shell hens were used as the experimental animals. Tissue samples including heart, liver, spleen, lung, kidney, duodenum, pancreas, ovary, and pectoral muscles of eight 30-week-old Lushi green-shell hens were collected at −80 °C after liquid nitrogen freezing. Liver samples of 5, 15, 20, 30, and 35-week-old Lushi green-shell hens were collected and stored at −80 °C after liquid nitrogen freezing.

Thirty-two healthy 10-week-old hens were randomly divided into four groups, with eight birds in each group. They were feed in the same environment with free access to feed and water. 17β-estradiol was dissolved in anhydrous ethanol. The birds in the three experimental groups were intramuscularly injected with 17β-estradiol at the final concentration of 0.5, 1.0, and 2.0 mg·kg^−1^ body weight, respectively. The birds in the control group were administered an equal volume of anhydrous ethanol. After treatment for 12 hours, chickens in each group were slaughtered, and the liver tissues were collected and stored at −80 °C after liquid nitrogen freezing.

### 4.3. Target Gene Prediction of gga-miR-221-5p and Bioinformatics Analysis 

The miR-221-5p mature sequences of different species including *Gallus gallus, Capra hircus, Homo sapiens, Columba livia, Rattus norvegicus, Mus musculus, Sus scrofa, Cricetulus griseus, Macaca mulatta, Danio rerio, Dromaius novaehollandiae, Oryctolagus cuniculus, Crocodylus porosus, Gadus morhua*, and *Xenopus laevis* were downloaded from the miRBase database (http://www.mirbase.org/), and used to analyze the conservatism. The target genes of gga-miR-221-5p were predicted as shown in Figure S1. In detail, the TargetScan 7.1 (http://www.targetscan.org/vert_71/) and miRDB (http://www.mirdb.org/) were used to predict the target genes of gga-miR-221-5p. The potential targets were obtained based on the intersection between the predicted target genes and the differentially upregulated genes in the liver transcriptome of the pre-and peak-laying hens [30]. The genes related to lipid metabolism including *BTG2, CYP7A1, ADIPOR2, AGPAT3, SQLE*, and *ELOVL6* were selected for further experiment. 

The minimum free energy (MFE) of the gga-miR-221-5p binding to the candidate target genes was calculated using hybrid online software (http://bibiserv.techfak.uni-bielefeld.de/rnahybrid). The ERE sequence matrix was obtained from JASPAR (http://jaspar.genereg.net/). The promoter sequences of gga-pri-miR-221-5p, *ELOVL6,* and *SQLE* were obtained from the NCBI database (https://www.ncbi.nlm.nih.gov/gene), then, they were used to analyze the ERE sites via the online software FIMO (http://meme-suite.org/tools/fimo).

### 4.4. Vector Construction

The 3′UTR fragments of *ELOVL6* and *SQLE* (including Xho I and Not I restriction endonuclease sites) including the binding sites of gga-miR-221-5p were cloned, respectively. Primers used for cloning were designed on the NCBI website (primer information in Table 1). The 3′UTR fragment including the wild-type gga-miR-221-5p binding region was amplified by PCR, and the 3′UTR fragment including the mutant-type gga-miR-221-5p binding region was amplified by overlap-PCR. PCR amplification program: 95 °C for 5 min; 35 cycles at 95 °C for 30 s, 60 °C for 30 s, and 72 °C for 30 s, followed by 72 °C for 10 min. The amplified products were purified and retrieved by using the DNA purification and recovery kit instructions (Tiangen, Beijing, China). Then, the restriction enzymes Xho I and Not I were used to digest the psiCHECK2 plasmid (Promega, Madison, WI, USA) and the retrieved target DNA fragments. Finally, the digested psiCHECK2 vector and target DNA fragments are ligated with T4 DNA ligase (NEB, Beijing, China). The four recombinant vectors were named Vector-Luc-ELOVL6-WT 3′UTR, Vector-Luc-ELOVL6-Mut 3′UTR, Vector-Luc-SQLE-WT 3′UTR and Vector-Luc-SQLE-Mut 3′UTR, respectively. All the recombinant vectors were confirmed by sequencing (BGI, Wuhan, China). The recombinant plasmids were extracted by using the EndoFree Plasmid Maxi Kit (Tiangen, Beijing, China), and stored at −20 °C until use.

### 4.5. Cell Culture and Treatment

LMH chicken hepatoma cells were cultured in DMEM/F12 medium containing 10% fetal bovine serum (FBS), 1% penicillin G (100 U/mL), and streptomycin (100 μg/mL), and cultured in an incubator containing 5% CO_2_ at 37 °C. When cells fusion reached 70% in the six-well plate, miR-221-5p mimics and miR-221-5p mimics NC were transfected to the cells using lip2000 (Invitrogen, Carlsbad, CA, USA) according to the manufacturer’s instructions. The cells were collected after 48 h.

When sterilized specific pathogen free (SPF) eggs hatched to 17 embryos, they were removed and placed on a clean bench. Then, we took out chicken embryos and collected liver tissue, washed the liver using D-Hank’s solution and cut it into pieces and digested it with collagenase IV. After digestion was completed, it was filtered through 100-mesh and 500-mesh cell sieves to obtain cell suspension, centrifuged for 5 minutes at 1000 r·min^−1^ and repeated three times, then, the supernatant was aspirated and resuspended by adding Williams’s medium, and hepatocytes were purified by Percoll gradient centrifugation. The purified hepatocytes were resuspended in Williams’s medium, planted in six-well plates at 5 × 10^5^ cells·mL^−1^, and placed in a 37 °C, 5% CO_2_ incubator. We changed the medium after 12 h, when the cell fusion reached 80%, we changed to serum-free medium for 6 hours, and then used 0, 25, 50, and 100 nM 17β-estradiol treatment, respectively. The cells were collected after 12 h.

Estrogen action was mediated by liganded ER. Different estrogen receptor antagonists were used to estimate the type of liganded ER that delivered the estrogen action. The most ERα-selective compound was methyl-piperidino-pyrazole (MPP). The ICI 182,780 and tamoxifen (Sigma-Aldrich, Shanghai, China) were the antagonists of ERα and Erβ, in addition, they could act as agonists on GPR30 due to their high affinity for it. They were dissolved with dimethyl sulfoxide (DMSO). When primary hepatocyte fusion reached to 80%, starvation was performed for 6 h using serum-free medium and treated with MPP (100 mg· L^−1^), ICI (100 mg· L^−1^), and TAM (100 mg· L^−1^) for 3 h, respectively; then, we added 100 nM 17β-estradiol for 12 h. In addition, an equal volume of absolute ethanol treatment for 12 h and 100 nM 17β-estradiol treatment for 12 h were used as the blank control and positive control, respectively. Each group used three replicates. Finally, primary hepatocytes were collected and stored at −80 °C until use. The experiment was repeated independently three times.

### 4.6. Dual Luciferase Reporter Assay

When LMH chicken hepatoma cells fusion reached 70% in the 24-well plate, the cells were transfected with different combinations, including gga-miR-221-5p mimics combined with Vector-Luc-ELOVL6-WT 3′UTR, gga-miR-221-5p mimics combined with Vector-Luc-ELOVL6-Mut 3′UTR, gga-miR-221-5p mimics NC combined with Vector-Luc-ELOVL6-WT 3′UTR, gga-miR-221-5p mimics NC combined with Vector-Luc-ELOVL6-Mut 3′UTR, gga-miR-221-5p mimics combined with Vector-Luc-SQLE-WT 3′UTR, gga-miR-221-5p mimics combined with Vector-Luc-SQLE-Mut 3′UTR, gga-miR-221-5p mimics NC combined with Vector-Luc-SQLE-WT 3′UTR, and gga-miR-221-5p mimics NC combined with Vector-Luc-SQLE-Mut 3′UTR, respectively. Three biological repeats were performed in each group. Each group was set three replicates. After 48 h, the activity of the double fluorescence was detected according to the kit instructions (Promega, Madison, WI, USA).

### 4.7. Intracellular Triglyceride and Cholesterol Detection

To determine the effect of gga-miR-221-5p on the contents of intracellular TG and TC, gga-miR-221-5p mimics and inhibitors were transfected into LMH chicken hepatoma cells for 48 hours, respectively. The cells were washed twice with PBS and lysed with lysate according to the kit. Then, the supernatant of cell lysate was incubated at 70 °C for 10 minutes at room temperature and centrifuged at 2000× *g* for 5 minutes; finally, the supernatant was used to measure intracellular TG and TC contents according to Cell TG and T-CHO ELISA kit instructions (Applygen, Beijing, China), respectively. The total protein content in cells was determined according to the instructions of the Bradford Protein Quantification Kit (Applygen, Beijing, China).

### 4.8. cDNA Synthesis and Quantitative Real-Time PCR (qRT-PCR)

The total RNA of tissues and cells was extracted with the Trizol reagent according to the manufacturer’s instructions (Takara, Kyoto, Japan). The RNA quality was detected by agarose gel electrophoresis and ultraviolet spectrophotometer and stored at −80 °C According to the instructions of the reverse transcription kit (Takara, Kyoto, Japan), random primers were used for reverse transcription to obtain cDNA for mRNA amplification. The miRNA specific hairpin primers synthesized by RiboBio were used for reverse transcription to obtain cDNA for miRNA amplification.

The sequence information of qRT-PCR primers was listed in Table 2. All primers were synthesized by Shangya except gga-miR-221-5p primers, and gga-miR-221-5p primers were synthesized by RiboBio. The qRT-PCR was carried out according to the instructions of the QuantiFast SYBR Green PCR kit (Takara). The *β-actin* and U6 genes served as the internal reference genes of mRNA and miRNA quantification, respectively. Each reaction was repeated three times. The 10 μL qRT-PCR reaction system consisted of 2 × QuantiFast SYBR Green Master Mix 5 μL, 0.5 μL each of the upstream and downstream primers (10 μM), 1 μL of cDNA (about ng), and 5 μL RNase-free water. The normal qRT-PCR reaction procedure was as follows: pre-denaturation at 95 °C for 5 min; denaturation at 95 °C for 30 s, 60 °C for 30 s, 72 °C for 30 s for 40 cycles, and 72 °C for 10 min. The gga-miR-221-5p qRT-PCR reaction procedure was as follows: pre-denaturation at 95 °C for 3 min, denaturation at 95 °C for 12 s, 60 °C for 40 s, 72 °C for 30 s for 40 cycles, and 72 °C for 10 min.

### 4.9. Statistical Analysis

The gene relative expression level was analyzed using the 2^−ΔΔCt^ method. The data were presented as the mean ± standard error. A one-way analysis of variance (ANOVA) and *t*-test analysis of SPSS 20.0 software were performed to analyze the difference significance. The Duncan method was used to test the statistical significance of the differences among the groups. *p* < 0.05 indicated a significant difference. The graphs were drawn by using GraphPad Prism 5.0 software.

## Figures and Tables

**Table 1:** Primer sequences used for dual-luciferase reporter vector construction.

Primer Name	Primer Sequence (5’~3’)
*ELOVL6*-WT	F: ccgCTCGAGACTCTTGCCAGCGAGCGGCCC
	R: atttGCGGCCGCAGCTAAGCCATTGCA GCAAAATC
*ELOVL6*-overlap	F:CTGTGCCTCTGCACGCACCTGCCAGGTTGGGCGAAGATGTGCCAAGA
	R:TCTTGGCACATCTTCGCCCAACCTGGCAGGTGCGTGCAGAGGCACAG
*SQLE*-WT	F: ccgCTCGAGCCAAGGGGATGTGACTGGAC
	R: atttGCGGCCGCAGTGATGCACTCTGCAATGGAT
*SQLE*-overlap	F:CGGAAGGAAGTGTTGACGTTGCCAGGTCTGTCAGAATTTCATTCCTT
	R:AAGGAATGAAATTCTGACAGACCTGGCAACGTCAACACTTCCTTCC

**Table 2:** Primer sequences used for qRT-PCR.

Gene	Primer Sequence (5′~3′)	Annealing Temperature (°C)	Product Length (bp)	Accession Number
*BTG2*	F: GCTCGCAGAGCACTACAAACA	60	127	XM_418053.6
	R: GAGTCCGATCTGGCTAGCTG			
*CYP27A1*	F: GTGGACACGACCTCCAACAC	60	148	XM_422056.6
	R: GCATCGGCATCTTGGGGATA			
*ADIPOR2*	F: GGGAGGCGGTAGCGATTG	61	121	NM_001007854.1
	R: GATTATGCGTTGGCTCTGGC			
AGPAT3	F: GTCTCTCCATGACCGCTGAG	59	237	XM_004934600.3
	R: AACTGTCCTTTCTTTGTGCCG			
*SQLE*	F: GAATTGTTGCAGCCTGGTGG	60	241	NM_001194927.1
	R: ATTTTGCATTGGGCTCTGCC			
*ELOVL6*	F: GTGGATGCAGGAGAACTGGAAG	60	80	NM_001031539.1
	R: TTAGGTGCCGACCACCAAAT			
*β-actin*	F: GAGAGAAGATGACACAGATC	60	116	NM_205518.1
	R: GTCCATCACAATACCAGTGG			

## Supplementary Materials

The following are available online at https://www.mdpi.com/1422-0067/21/5/1624/s1.

## Abbreviations

ELOVL6	elongase of very long chain fatty acids 6
SQLE	squalene epoxidase
TG	Triglyceride
TC	Total cholesterol
VLDL	Very low density lipoprotein
MicroRNAs	miRNAs
WT	Wild-type
Mut	Mutation
ApoVLDL II	apovitellenin very low density lipoprotein Ⅱ
ERE	estrogen responsive elements
ER	estrogen receptor
miR-221-5p mimics NC	miR-221-5p mimics negative control
AP1	activator protein-1
