# Supplementary material for: Estrogen Abolishes the Repression Role of gga-miR-221-5p Targeting ELOVL6 and SQLE to Promote Lipid Synthesis in Chicken Liver
Source: Int J Mol Sci. 2020 Feb 27;21(5):1624. doi: 10.3390/ijms21051624 (PMC7084605; doi:10.3390/ijms21051624)
Supplement: Supplementary file 1 [file ijms-21-01624-s001.pdf]

International Journal of *Molecular Sciences*

MDPI

Article

Estrogen Abolishes the Repression Role of gga-miR-221-5p Targeting *ELOVL6* and *SQLE* to Promote Lipid Synthesis in Chicken Liver

Ding-Ding Zhang [1], Dan-Dan Wang [1], Zhang Wang [1], Yang-Bin Wang[1,2,3], Guo-Xi Li[1,2,3], Gui-Rong Sun[1,2,3], Ya-Dong Tian[1,2,3], Rui-Li Han[1,2,3], Zhuan-Jian Li[1,2,3], Rui-Rui Jiang[1,2,3], Xiao-Jun Liu[1,2,3], Xiang-Tao Kang[1,2,3], Hong Li [1,2,3]*

[1] College of Animal Science and Veterinary Medicine, Henan Agricultural University, Zhengzhou 450002, China; 15736702905@163.com (D.-D.Z.); wdd13938406174@163.com (D.-D.W.); wangzh19930124@163.com (Z.W.); ybwang2008@henau.edu.cn (Y.-B.W.); liguoxi0914@henau.edu.cn (G.-X.L.); grsun2000@126.com (G.-R.S.); ydtian111@163.com (Y.-D.T.); rlhan@henau.edu.cn (R.-L.H.); lizhuanjian@henau.edu.cn (Z.-J.L.); jrrcaas@163.com (R.-R.J.); xiaojun.liu@henau.edu.cn (X.-J.L.); xtkang2001@263.net (X.-T.K.); lihong19871202@163.com (H.L.)

[2] Henan Innovative Engineering Research Center of Poultry, Zhengzhou 450002, China

[3] International Joint Research Laboratory for Poultry Breeding of Henan, Zhengzhou, 450002, China.

* Correspondence: lihong19871202@163.com (H. L)

Received: 3 January 2020; Accepted: 24 February 2020; Published: date

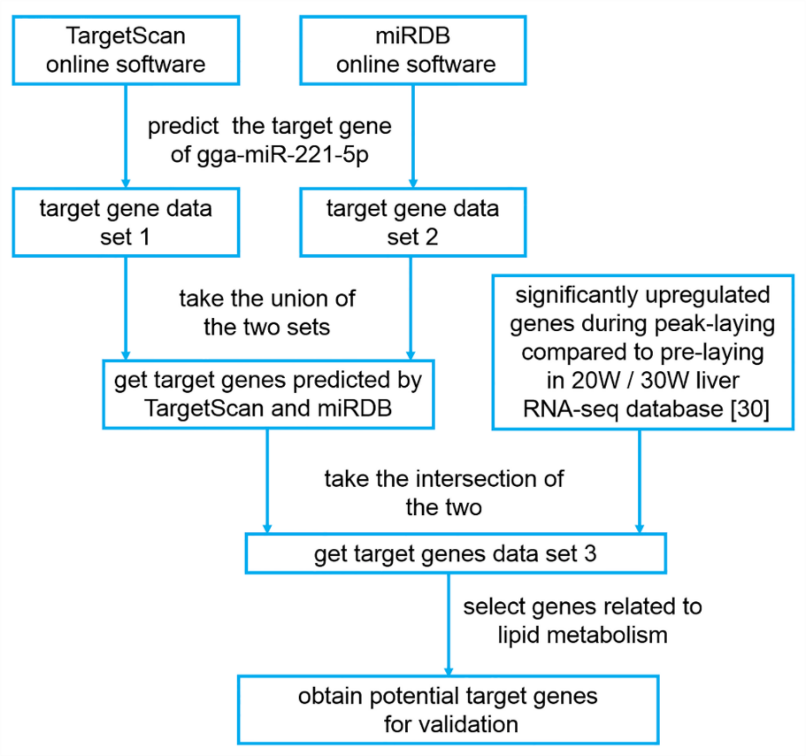

Supplementary Figure S1. Flow chart of gga-miR-221-5p target gene screening.
